# Quantitative determination of minerals and anthropogenic particles in some Polish peat occurrences using a novel SEM point-counting method

**DOI:** 10.1007/s10661-013-3561-0

**Published:** 2014-01-19

**Authors:** Beata Smieja-Król, Barbara Fiałkiewicz-Kozieł

**Affiliations:** 1 Department of Geochemistry, Mineralogy and Petrography, Faculty of Earth Sciences, University of Silesia, Będzińska 60, 41-200 Sosnowiec, Poland; 2 Department of Biogeography and Palaeoecology, Faculty of Geographical and Geological Science, Adam Mickiewicz University, Dzięgielowa 27, 61-680 Poznań, Poland

**Keywords:** Peat, Dust deposition, SEM, Fly ash particles, Minerals

## Abstract

A method is proposed for determining the mineral composition of peat using scanning electron microscope. In an illustrative example, five groups of particles occurring in amounts of >0.05 % are distinguished in peat from Puścizna Mała bog in the Carpathian foreland, Poland. These are spheroidal aluminosilicate particles (SAP), feldspars, nondescript aluminosilicates (mainly clays), silica (quartz and opaline silica), and Fe(hydro)oxides. Two more site-specific groups (barite and ZnS) are distinguished in highly polluted fens (Bagno Bruch and Bagno Mikołeska) near a zinc smelter in Upper Silesia. At Bagno Bruch, peat contents of predominantly authigenic ZnS microspheroids range up to 1.1 %. SAP originating from coal-burning power stations account for maximum concentrations of <21–39 % of the inorganic fraction in the studied mires. SAP concentrations vary with depth, and mean spheroid diameters with distance from emission sources. A distinct feature of SAP is their common enrichment in Ti what questions the use of Ti as a proxy for soil dust in fly ash polluted bogs. As amounts of anthropogenic magnetic spherules, less abundant than SAP in all mires, relate to water table level position, they are unsuitable as tracers of air pollution. The proposed method is recommended for application with peats having ash contents > ~4 %.

## Introduction

Ash remaining after the burning of peat at 550 °C is important in the characterization of peatland type and trophy status. In ombrotrophic bogs, ash contents that reflect atmospheric-dust deposition are generally very low, between 1 and 3 % (Steinmann and Shotyk 1997). In fens, mineral matter is also contributed by surface runoff or precipitated directly from metal-rich groundwater (Syrovetnik et al. 2007); amounts are generally >5 % (Steinmann and Shotyk 1997).

The mineral composition of ombrotrophic peat is rather simple and uniform with depth. For example, the ash from Etang de la Gruère peat bog (Jura Mountains, Switzerland) contains mainly quartz (60–90 %) and opaline silica (30–70 %) with lesser amounts of feldspar (5–15 %) and layered silicates, mainly muscovite (5–15 %) (Steinmann and Shotyk 1997). Mineral contents diminish over time as less resistant minerals are removed by weathering (Bennett et al. 1991; Le Roux et al. 2006; Le Roux and Shotyk 2006). However, most European bogs, even those in remote sites, show a 3- to 5-fold increase in ash content in subsurface layers relative to deeper sections, giving a characteristic C-shaped ash distribution curve (West et al. 1997; MacKenzie et al. 1998; Martinez-Cortizas et al. 2002; Franzén 2006; Le Roux et al. 2006; Fiałkiewicz-Kozieł et al. 2011). The subsurface increases are the result of anthropogenic dust contributions, mainly from agriculture and industry (Shotyk 1988). Adjacent to industrial centers, e.g., in peat bogs close to coal-based power stations in the northern part of the Czech Republic, ash contents < ~17 % characterize the subsurface layer (Zuna et al. 2012). Peak ash contents often correlate with elevated heavy metal concentrations and typically reflect industrial activities in the 1970–1980s (MacKenzie et al. 1998).

A small number of studies have shown that along with increases in content, mineral compositions also change in uppermost peat layers. In the upper parts of ombrotrophic peat bogs, the occurrence of ferromagnetic particles originating primarily from coal burning has been documented using magnetic susceptibility methods (Williams 1992; Strzyszcz and Magiera 2001; Zuna et al. 2012). Punning and Alliksaar (1997) showed that the fly-ash particle distribution curve for the upper part of a peat bog near Tallinn, Estonia, correlates well with the history of fuel consumption and air pollution. Various exotic anthropogenic particles, often containing heavy metals (Fe, Zn, Pb, Cu, and Sn), have been imaged in peats by Rausch et al. (2005), Le Roux and Shotyk (2006), and Smieja-Król et al. (2010) using scanning electron microscope (SEM).

Though a variety of methods have been used to analyze peat mineral compositions, quantitative evaluations are rare. Examples are bulk peat samples, or samples after removal of the organic constituents, examined mainly by XRD (e.g., López-Buendía et al. 2007). Specific inorganic fractions have been quantified using optical microscopy, e.g., sand grains in Sweden peatlands (Björck and Clemmensen 2004), carbonaceous- and inorganic fly-ash particles (Punning and Alliksaar 1997) and tephra shards (Swindles et al. 2010).

Quantitative data derived using different techniques can be difficult to compare. Optical-microscop techniques tend to fall short when it comes to the finest fraction (e.g., Punning and Alliksaar 1997; Steinmann and Shotyk 1997). XRD studies fail to detect non-crystalline inorganic components and, thus, predominantly glassy inorganic fly-ash particles and tephra shards remain undetected or under-represented.

The present study documents changes in the inorganic-fraction compositions in sections that range from untouched bottom-peat layers to uppermost anthropogenically affected layers in (a) an ombrotrophic bog sited 60–100 km from any larger industrial centre and (b) two poor fens adjacent to an urban industrial agglomeration. For the upper layers, the contribution of anthropogenic particles relative to geogenic mineral phases is determined. In doing so, a method that enables the quantitative determination of amounts and compositions of inorganic particles using SEM is presented and its potential evaluated.

## Site description

**Fig. 1:**
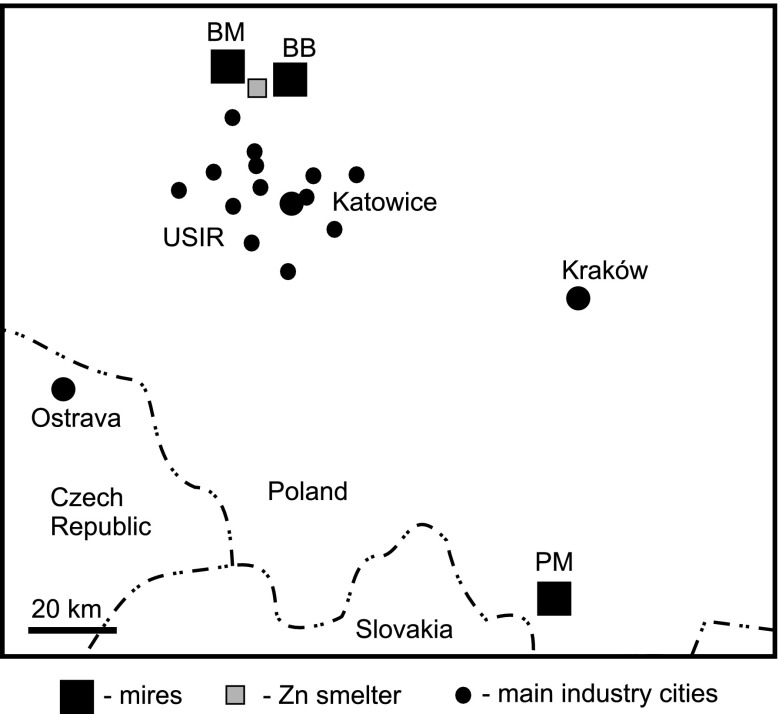
Location of the studied mires in the southern part of Poland. *BB* Bagno Bruch, *BM* Bagno Mikołeska, *PM* Puścizna Mała, *USIR* Upper Silesia Industrial Region

The Puścizna Mała peat bog lying in the Orava-Podhale depression of the Carpathian foreland is a part of Orawsko-Nowotarskie ombrotrophic peatlands, the largest peatland complex in southern Poland (Fig. 1). The core material analyzed (PM1) was collected from the surface of peat bog dome as a peat monolith (130 × 20 × 20 cm) in the early summer of 2006 using a wooden box (Tobolski 2000). Palynological analyses, radiocarbon and Pb dates, and the bulk composition of the peat core (bulk density, ash value, carbon content, sulfur, and nitrogen) have been presented elsewhere (Kołaczek et al. 2010; Fiałkiewicz-Kozieł et al. 2013). For the present study, the core was sliced into 1.5 (top 15 cm) and 3 cm (deeper part) pieces.

Bagno Bruch and Bagno Mikołeska are small mires located in an inland dune field developed along the Mała Panew Valley during the Late Pleistocene (Szczypek 1977) in the northern part of the Silesian Upland (Fig. 1). The dune field is part of the so-called European sand belt (Zeeberg 1998). Bagno Bruch was characterized in detail by Smieja-Król et al. (2010). Bagno Mikołeska occurs in a small (5 ha) inter-dune depression in which the peat layer is >2 m thick. The mire vegetation belongs to the C*aricetum lasiocarpae sphagnetosum fallacis* association, *Juncus effusus—Sphagnum fallax* and *Eriophorum vaginatum—S. fallax* communities (Konior 2004). The upper 45 cm of peat is dominated by *sphagnum* species, the lower part is predominantly herbaceous (Fiałkiewicz-Kozieł et al., unpublished data). Both mires are supplied mainly by rainwater with some probable groundwater addition. There is no surface-water input.

Both mires lie close to the Upper Silesia Industrial Region (USIR), a vast industrial complex involving, till present, coal mining, iron- and steel production and energy and, in the past, large-scale Zn–Pb mining and smelting. Currently, four coal-based power plants with a generating capacity of >1,000 MW operate there. Approximately 10 km south of Bagno Mikołeska and west of Bagno Bruch, a Zn–Pb smelter, the last in the region, has operated since 1968.

Using a Wardenaar corer (Wardenaar 1986), two cores were taken of the Bagno Bruch mire, BB1 from the centre and BB2 from a site closer to the edge, and one (BM1) from the centre of the Bagno Mikołeska mire. In the laboratory, all were sectioned into 1 cm slices with a stainless steel knife.

## Laboratory methods

A small portion of each slice (~1 cm^3^) was taken for mineralogical analysis and air dried. The sample was further divided into two subsamples and part of each was gently homogenized using a corundum mortar and pestle to prevent any addition of silica. A thin layer of each homogenized sample was fixed to a double-sided 9 mm carbon tab, placed on an aluminum stub and carbon coated prior to analysis. Care was taken that the homogenized material was firmly glued to the carbon tab and that the roughness of the surface was minimized.

The analyses were carried out using an environmental scanning electron microscope, Philips XL 30, coupled to an energy dispersive X-ray analyzer system (EDS). A Centaurus back-scattered electron (BSE) detector was used for imaging. The BSE detector yielded the phase contrast necessary to distinguish between inorganic (bright) and organic (dark) phases. The accelerating voltage was 15 kV and 10 mm the working distance.

A point counting method was used to quantify the inorganic fraction. The method is used routinely in coal petrology to determine maceral-group compositions under optical microscope (Taylor et al. (1998); ISO 7404–3 (1994). Recently, the method has been applied in studies of maceral groups in coal waste (Misz-Kennan and Fabiańska 2010) and of carbonaceous particles in fly ash and slags (Misz 2002) where the components of interest constituted a minor fraction. As the volume of the inorganic fraction is low in peat, the number of counted points was increased from the coal-standardized method of 500 points up to 2,000 points for each depth interval—a compromise embracing the time needed to do an analysis and the statistical demands of small volume fractions. The optimum number of grid test points is a function of the volume fraction to be measured and is determined by the equation *P* = 3/*V*_V_, where *V*_V_ is the volume fraction (Underwood 1970). The 2,000 counting points gives the method reliability at a level of 0.15 %. The detection limit, defined as one “hit” on a phase of interest in the point grid, equals 0.05 %.

Analysis was undertaken at ×10,000 magnification. Fields of view were moved in uniform increments and, in each, the phase under the crosswire noted. The spacing of points was ~140 μm. Elemental compositions of inorganic particles were determined by EDS. With very small particles, magnification was increased to obtain the most reliable spectrum. Particle-surface irregularity and predominantly small particle sizes make the EDS data semi-quantitative. Thus, elemental ratios in the spectra were treated with caution when identifying inorganic phases. The detection limits in EDS measurements are about 0.1 wt.%.

The 2000 points were counted in four separate grid fields of 500 counts, two for each of two stubs. Standard deviations were calculated based on the number of particles counted in each field (4 × 500). Additionally, the sizes of the counted particles were determined in two profiles (PM1 and BM1) by measuring two orthogonal dimensions across each grain. Size is presented as the root-mean-square size, r.m.s. = {*a* ^2^ + *b* ^2^}^1/2^, where a and b are the two orthogonal grain dimensions (Rietmeijer and Janeczek 1997).

Ash contents (AC) were obtained by burning 1 g of peat sample at 550 °C. AC = weight of sample burned at 550 °C/weight of sample dried at 105 °C*100 %.

Rainwater collected during winter 2010–2011 in 1.5 L polyethylene bottles was filtered through 0.45 membrane filters. Dust deposited on the filters was analyzed by XRD using a PANalytical device `X PERT PRO PW 3040/60 (CoKα radiation), equipped with an X'Celerator detector. Measurement parameters were: voltage, 45 kV; current, 30 mA; counting time, 300 s pulse; and a scanning speed of 0.01°2*θ*/min. Minerals were identified using the ICSD database (version 2007/12).

## Results

### Ash- and inorganic-particle contents in the peat

**Fig. 2:**
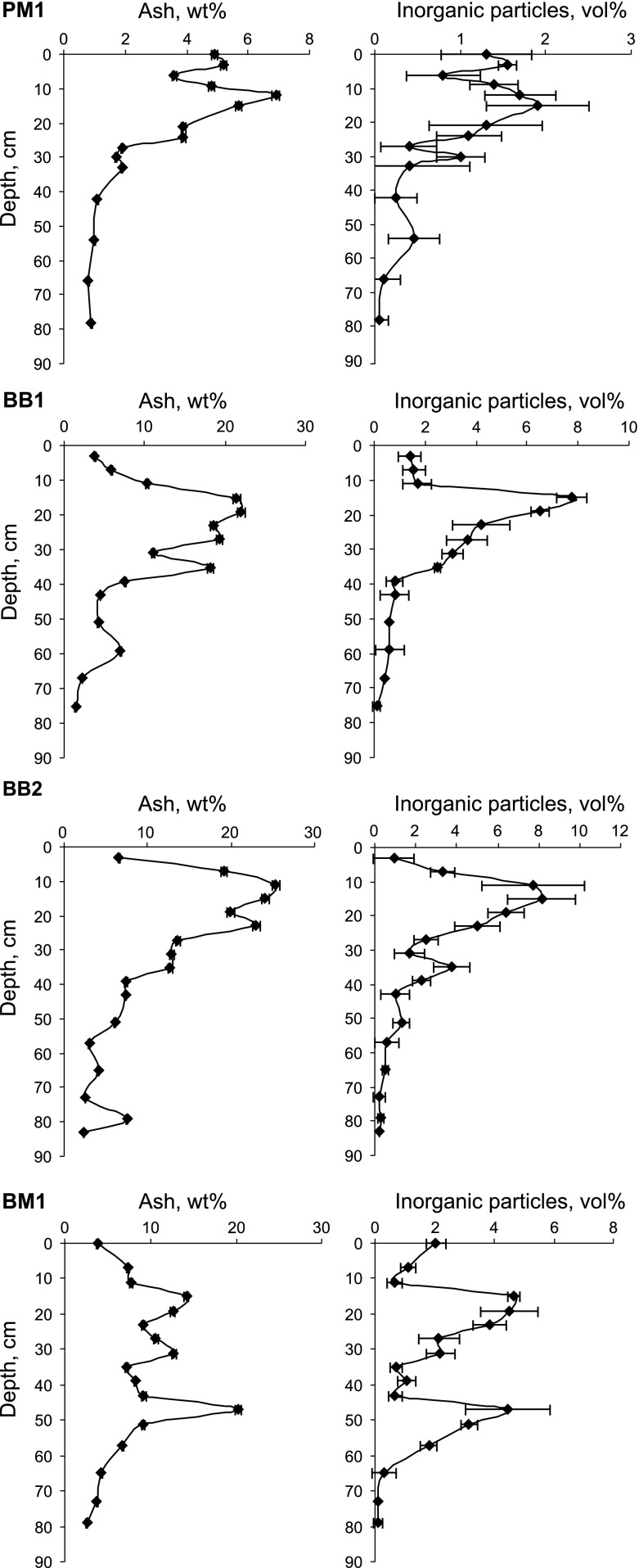
Ash contents and amounts of inorganic particles in peat profiles

Pronounced variations in both the ash- and inorganic-matter contents are evident between profiles and between deeper- and near-surface peat layers. These variations reflect the trophy status of the mires and dust deposition loads. The latter are much higher in industrialized Upper Silesia than in the Orawsko-Nowotarskie region (Fig. 2).

The ash content in PM1 is ~1 % in the lower layer (Fig. 2). It gradually increases from about 42 cm to a maximum of 6.9 % in the 12–13.5 cm layer. Nearer the surface, the ash content is lower (4.9 %). Ash contents follow a similar trend in the BB1 and BB2 profiles although contents are slightly higher in BB2. The lower sections of BB1 and BB2 (43–84 cm) have ash contents between 1.4 and 7.6 % while ash contents in the upper layer (0–40 cm) are markedly higher and range between 3.8–25 %. Maximum ash values are similar for both profiles, being 22 % in the 19–20 cm layer in BB1 and 25 % in the 11–12 cm layer in BB2. The BM1 shows a much more complex ash distribution. In five samples from the deeper section (51–79 cm), ash contents vary between 2.6–9.1 %. The maximum ash value of 20 % occurs at a depth 47–48 cm, decreases to 7 % at 35-36 cm before rising, in the upper peat layer, to a highest value of 14 % at 15–16 cm. As in PM1, the uppermost BM1 peat layer provides relatively little ash. In subsurface peat, ash contents vary between 3.8–6.5 % in Bagno Bruch and reach 3.8 % in Bagno Mikołeska.

At Puścizna Mała, amounts of inorganic particles are low along the entire PM1 profile (Fig. 2) being between <0.05 and 0.7 % in the lower layer and increasing slightly upward to a maximum of 1.9 % at 15–18 cm. Inorganic particles account for 1.3 % of the uppermost layer (0–3.5 cm). The inorganic particle content which in the lower layers at Bagno Bruch ranges between 0.05 and 1.3 % rises, from 40 cm upward, to a maximum of ~8 % at 15–16 cm in both BB1 and BB2 before lessening towards the surface to values of 1.4 in BB1 and 0.95 % in BB2. In Bagno Mikołeska, the inorganic-particle contents range between <0.05 and 4.7 % with two distinct maxima of 4.5 % at 47–48 cm and 4.7 % at 15–16 cm with as at Bagno Bruch, lower numbers in the subsurface layer (Fig. 2).

For all samples analyzed (*n* = 64), ash- and inorganic-particle contents display a strong positive linear correlation (*R* ^2^ = 0.80). Treating the profiles separately, the lowest value of the correlation coefficient characterizes BM1 (*R* ^2^ = 0.60; *n* = 17). This relatively low value probably reflects the fact that herbaceous plants predominate in the lower part of this profile and, thus, ash contents are less related to mineral contents.

### Mineralogical composition

**Fig. 3:**
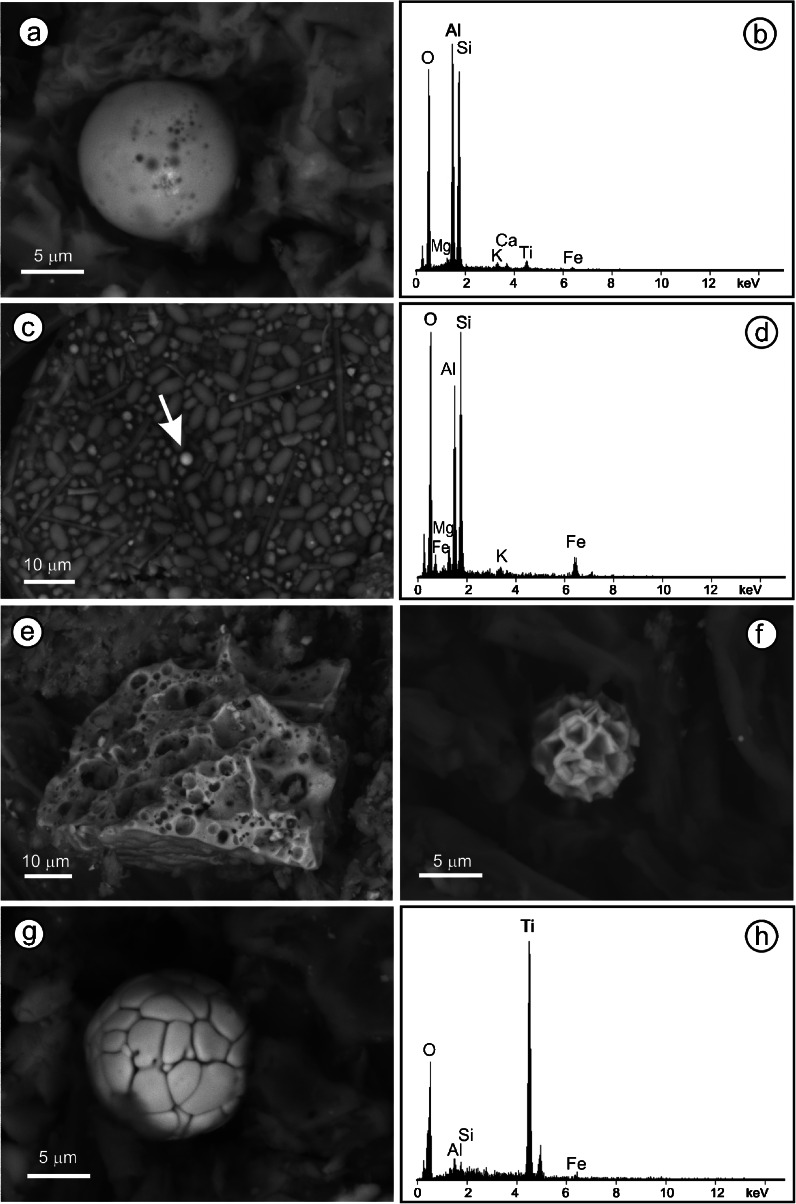
BSE images of anthropogenic particles. **a** Typical SAP and **b** the corresponding EDS spectra showing Ti enrichment in the particle, PM1, depth, 6–7.5 cm; **c** testate amoeba with incorporated spherical fly ash particle (indicated by *arrow*), BB1, 13–14 cm; **d** EDS analysis of the spherical particle in (**c**); **e** porous aluminosilicate aggregate, BM1, 15–16 cm; **f** spherical Fe oxide, PM, 9–10.5 cm; **g** spherical Ti oxide; and **h** the corresponding EDS analysis, BB2, 15–16 cm

The SEM-BSE investigation revealed that the inorganic particles in the cores fall into five groups based on elemental composition and morphology, namely, feldspars, spheroidal aluminosilicate particles (SAP), nondescript aluminosilicate particles, silica and Fe (hydro)oxides (Fig. 3). Additionally, barite (BaSO_4_) and ZnS were shown to constitute site-specific fractions in the Upper Silesian mires (Bagno Bruch and Bagno Mikołeska). Particles in amounts too low to be quantified (<0.05 %) are deemed accessory minerals/phases.

SAP in the peats reflect dust deposition. They formed at high temperatures, mainly during coal combustion, and are a dominant constituent of fly ash. The spheroidal shape and smooth surface of smaller particles (Fig. 3a, c), and the spheroidal shape and vesicular texture of larger grains, allows their discrimination from natural particles. In addition, larger (>10 μm) SAP cenospheres are commonly hollow. Even if fragmented, SAP are recognizable (Fig. 3e). Chemically, they are aluminosilicates with K, Mg, Na, and traces of Fe and Ca (Fig. 3d), very close to or undistinguishable from other aluminosilicate phases present. Common enrichment in Ti is a distinct feature of SAP (Fig. 3b). SAP with Mn, Pb, Zr, or Zn are rare in any of the mires.

Feldspars (plagioclase and potassium feldspar) are the only aluminosilicates distinguishable solely on the basis of chemical composition. As such, they constitute a separate group.

**Fig. 4:**
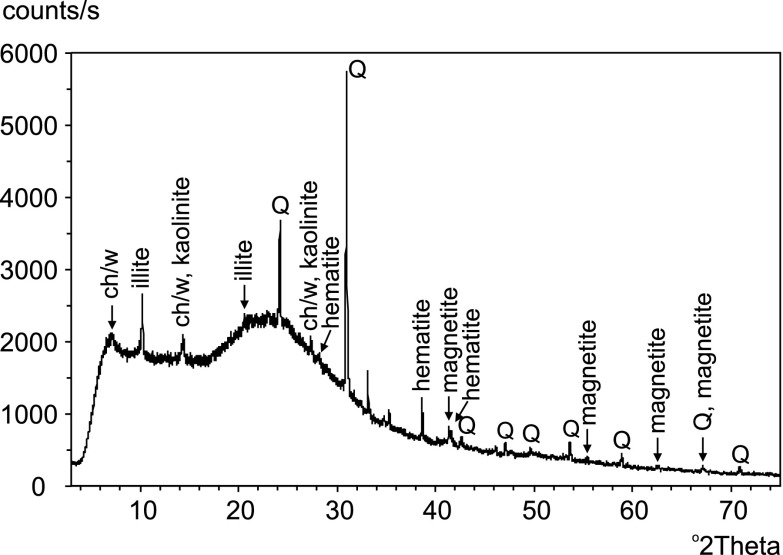
X-ray diffraction pattern of dust deposited at Bagno Mikołeska during the winter of 2010–2011

Nondescript aluminosilicate particles are a group of dust-deposited particles with compositions dominated by Si and Al with additional Na, K, Mg, Ca, and Fe. As most are very small (1–4 μm) and irregular particles, precise identification is not always possible. Nevertheless, shape, cleavage and weathering features indicate that they are mainly clays and to a lesser extent, other layered aluminosilicates (micas). XRD analysis confirms significant recent depositions of illite, chlorite/vermiculite minerals and kaolinite (Fig. 4) in Silesian mires.

The silica group includes detrital quartz grains and opaline silica of biogenic origin. As quartz is common in fly ash (e.g., Vassilev and Vassileva 1996), quartz grains that emanated from power stations are to be expected in upper peat layers. Biogenic silica is represented mostly by phytoliths distinguished by morphology and regular distribution inside plant cells. Silicious bio-mineralizing testate amoeba are rarely seen (Fig. 3c) as are fragments of diatom tests. In deeper layers, most silica grains are small, angular to rounded and lack distinct morphologies. Larger (>10 μm) quartz grains of undoubted detrital origin are relatively rare in all profiles except for the interval 47–52 cm in profile BM1.

Fe(hydro)oxide particles are dust deposited. When spherical (Fig. 3f), their origin is similar to that of SAP. Spheroidal Fe oxides (maghemite, magnesioferrite, magnetite, and hematite) are the dominant morphology in dust from coal and lignite combustion and coke production (Magiera et al. 2011). Fe(hydro)oxides with nonspheroidal shapes may be either detrital or anthropogenic. The Fe(hydro)oxides may contain Mg, Zn, and Mn, Ca and Ni, less commonly, Ti, and Cu.

ZnS particles were found in amounts > ~0.05 % only in the Upper Silesian mires. They occur as irregular weathered particles deriving, most probably, from the nearby smelter and much more abundant, authigenic growths. The dust ZnS is, in some cases, enriched in Fe. The authigenic ZnS commonly contains Cd (< ~4 %). The authigenic ZnS grains are typically small (~1 μm) and spherical or semispherical if attached to organic material. Hundreds of these microspheroids may be aggregated in organic tissues (Smieja-Król et al. 2010).

Barite occurs in amounts > ~0.05 % only in the Upper Silesian mires. In atmospheric dust, the barite derives from coal burning (Jabłońska et. al. 2001) and/or Zn-Pb ore smelting. Authigenic barite, as well-formed tabular crystals set in organic tissues, was found only in the Bagno Bruch fen. Some minute, needle-like grains may be artifacts of the peat drying.

### Distribution of inorganic particles with depth

**Fig. 5:**
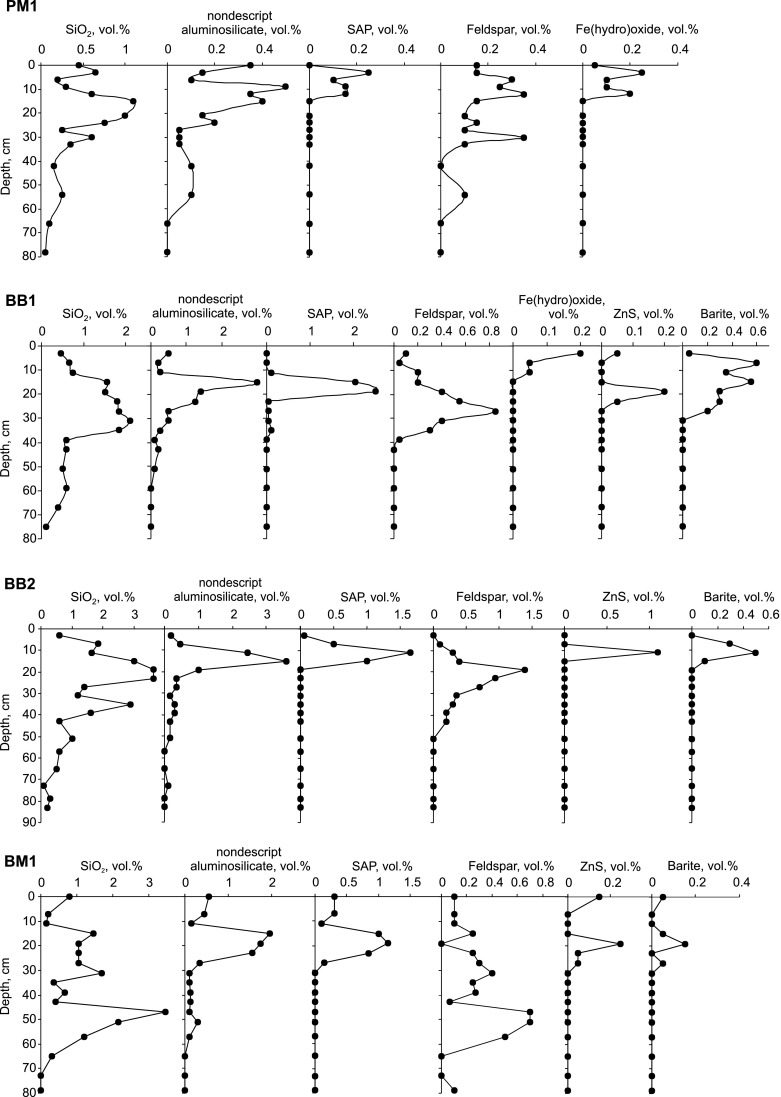
Mineral- and anthropogenic-particle distribution in the peat profiles
  From 13.5 cm upwards, SAP and Fe(hydro)oxide start to contribute significantly to inorganic-particle contents. Here, SAP occurs in amounts ranging from 0.1 to 0.25 %. The maximum content at 3.0–4.5 cm represents 17 % of all inorganic particles there. From 13.5 cm upwards also, Fe(hydro)oxide occur in concentrations of >0.05 %, reaching a maximum of 0.25 % at 3–4.5 cm. Spheroidal and irregular forms are equally represented.
  Accessory phases of detrital origin (<0.05 %) include ilmenite, zircon, Ti oxides, and REE phosphates). These occur sparingly, a few particles per sample, throughout the PM1 profile. Small (<10 μm) grains of ZnS, barite, wolframite, weathered particles of Pb, Zn, Sb oxides, Cu–Zn alloy, and Cu sulfide are likely of anthropogenic origin; these occur from 0 to 24 cm, extending below the interval of SAP abundance. Gypsum, precipitated in situ and impregnating plant tissues, was found at 15-18 cm.
- Bagno Bruch
  The inorganic particles follow similar distribution trends in both profiles (Fig 5), e.g., BB2 contains visibly more silica and feldspars, consistent with the higher ash content and to its location closer to the mire border. The SAP concentration is higher in BB1.
  The mineralogy of the peat below 40 cm is simple and relatively constant; in both profiles, silica dominates, the contribution of nondescript aluminosilicates is low and that of other groups below detection. In the upper peat layers, particle concentrations rise significantly. Increasing silica and feldspar precedes the appearance of anthropogenic SAP and an increase in nondescript aluminium silcates. Feldspar, at maximum concentration, accounts for ~24 % of the inorganic fraction, with K feldspar dominating over plagioclase in both profiles.
  SAP constitute an important addition to the upper peat layer, matching or exceeding the abundance of silica (Fig. 5). In BB1, the highest SAP concentration (2.5 %; 39 % of the inorganic fraction) occurs at 19–20 cm. In BB2, SAP particles are restricted to the 7–16 cm layer with the maximum concentration (1.65 %; 21 % of the inorganic fraction) occurring at 11–12 cm. They decrease to 0–5 % in the subsurface layer (3–4 cm) in both profiles.
  ZnS particles follow the SAP distribution in both Bagno Bruch profiles (Fig. 5). Secondary processes, and differences in hydrological conditions, may serve explain any differences in amounts between the two. Only 0.2 % of these particles were detected in BB1 and 1.1 % in BB2—the reverse of the SAP pattern. Secondary processes may also be relevant to the distribution of barite, also prone to dissolution and authigenic re-precipitation. As coal combustion and smelting are the source of barite, its distribution might be expected to follow those of SAP and ZnS. However, barite is widely present between 7–28 cm in BB1, with a maximum (0.6 %) at 7–8 cm—above those of ZnS and SAP. In BB2, by contrast, the barite, SAP, and ZnS maxima coincide. In BB1, Fe(hydro)oxides contents are low (0.1–0.2 %) and restricted to 0–12 cm; in BB2, they are present (at 0.1 %) only in the uppermost (3–4 cm) peat.
  Galena (PbS) is a characteristic accessory phase in the Bagno Bruch mire. In the BB1 core, galena and ZnS particles (auhigenic and dust derived) occur together down to a depth of 24 cm whereas, though galena is relatively more abundant in BB2, it dominates over ZnS particles only in lower (17–24 cm) peat layers. Authigenic gypsum, in some cases associated with barite, occurs sporadically between 7 and 44 cm in BB1 and 27–28 cm in BB2. In the uppermost peats, complex oxides and/or alloys of Pb–Ti–Mn–Fe, Pb–Sn, and Pb–Sn–Sb together with gahnite (ZnAl_2_O_4_) and Zn silicate derive from the nearby smelter. Ti oxide is the most abundant heavy accessory everywhere.
- Bagno Mikołeska
  The distribution of particles in the upper peat layer of BM1 (Fig. 5) is similar to those in the Bagno Bruch profiles. High concentrations of SAP are seen between 15 and 24 cm and a maximum of 1.15 % (26 % of all particles) at 19–20 cm. In the 0–3.5 cm subsurface layer, SAP accounts for only 3 % of the inorganic fraction. Maximum contents of ZnS (0.25 %) and barite (0.15 %) also characterize the 19–20 layer. Feldspars reach a maximum (0.4 %) together with silica (1.7 %) in the 31–32 cm layer and, as at Bagno Bruch, K feldspar (64 %) is more abundant than plagioclase. Nondescript aluminosilicates are notable at 15-16 cm, slightly above the SAP-, ZnS-, and barite maxima. Increased silica (<3.45 %) at 47–48 cm correlates with a slight increase in feldspar (0.7 %) and nondescript aluminosilicates (0.3 %).
  In this fen, Fe(hydro)oxides are an accessory fraction. Nevertheless, individual, weathered grains occur up to a depth of 32 cm. Occasional, small (<5 μm) barite grains occur over a wide interval between 0 and 44 cm. Galena grains (1–8 μm) are occasionally found between 27 and 52 cm, i.e., generally below the main occurrence of predominantly authigenic ZnS.

- Puścizna Mała
  Up to 15 cm below the peat surface, the mineralogy is rather simple throughout the profile with only silica, feldspars and small ill-defined aluminosilicate particles occurring in amounts > ~0.05 % (Fig. 5). Silica is a dominant component throughout, comprising 23–80 % of the inorganic fraction. The aluminosilicate particles and feldspar occur in similar amounts with both increasing slightly from the 30 cm level upwards. In the entire profile, the former account for 5–38 % and feldspars for 8–38 % of the inorganic fraction. Amounts of K feldspar and plagioclase (mainly albite) are comparable.

### Particle size distribution

**Fig. 6:**
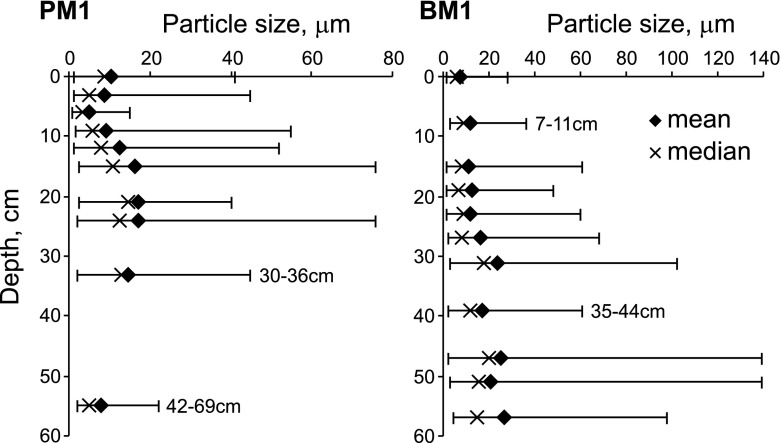
Particle size (min–max, mean, and median) variations in PM1 and BM1

Particle sizes range from 1 to 76 μm in the PM1 profile and from 1.3 to 139 μm in BM1 (Fig. 6). The average particle size, calculated for each depth interval separately, falls in a relatively narrow range between 5 and 18 μm in PM1 and 7 and 25 μm in BM1. Particles at <10 μm tend to dominate in the uppermost peat layers in both profiles. The median value lies between 3.5 and 9 μm in the depth range 0–13.5 cm in PM1 and between 5.8 and 8.8 μm in a depth range 0–28 cm in BM1. Larger particles are more common in deeper peat sections (Fig. 6).

**Fig. 7:**
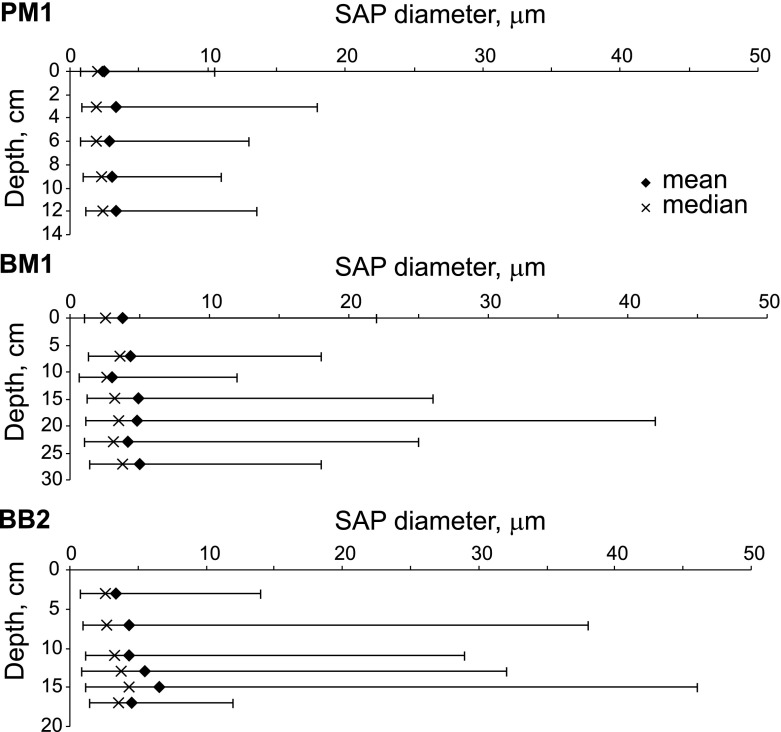
SAP diameter (min–max, mean, and median) variations in PM1, BM1, and BB2

The diameters of SAP and spheroidal Fe oxides, representing the anthropogenic fraction, were measured in PM1, BM1, and BB2 (Fig. 7). To obtain adequate numbers (*n* = 65) for each depth layer, all spherical particles in ×10,000 fields of view were measured. Crushed SAP fragments and large cenospheres of complex morphology were ignored.

In the cores, the depth interval in which SAP particles occurred in sufficient numbers differed from one to the other. Even where they amounted to <0.05 %, it was still possible to measure 65 spheres per sample in some cases. SAP occur within a relatively narrow depth range between 0 and 13 cm in PM1 and, in the Upper Silesian mires, between 0-18 cm in BB2 and 0–28 cm in BM1. The depth intervals of SAP occurrences are mostly related to varying peat accumulation rates. The top 19 cm of the Puścizna Mała core accumulated in 147 ± 15 years, corresponding to a core-averaged accumulation rate of 1.3 ± 0.1 mm/years (Fiałkiewicz-Kozieł et al. 2013), whereas the uppermost 18 cm of peat at Bagno Bruch accumulated in 61 ± 6 yrs at a rate between 2.3 and 3.5 mm/year—more than twice as fast (Smieja-Król et al. 2010).

Particles in the PM1 profile are smaller (average, 2.5–3.4 μm) than those in BM1 (3.0–5.0 μm) and BB2 (3.4–7.4 μm). However, only small differences in the averages with depth are evident. SAP median values, 1.9-2.4 μm in PM1 and 2.7–4.3 μm in BB2, increase slightly with depth in both. In BM1, both the median (2.6–3.8 μm) values and the averages are independent of depth. In both PM1 and BB2, Fe oxide spheres are markedly smaller in comparison to SAP. Between 0–13.5 cm in the PM1 core, average- and median values are 1.2–2.6 and 1.0–1.4 μm, respectively while, in the uppermost (3–4 cm) of BB2, the corresponding values are 2.4 and 1.6 μm.

## Discussion

### Ash contents and inorganic particle numbers in the peat

Ruling surface-water input out, the inorganic fraction reflects the amount of deposited dust in all of the peats studied. The inorganic-particle analysis provides quantitative data on temporal changes in atmospheric dust deposition even in mires not fully ombrothrophic. Only an increase in biogenic silica due to changes in plant composition can be expected in the fens. In the case of the Upper Silesian mires, as the groundwater comes from essentially nutrient-deficient fluvioglacial sands, precipitation of authigenic minerals due to increased element concentrations in pore water may be disregarded. Furthermore, authigenic minerals are easily distinguished from dust-deposited particles (e.g., Cabała et al. 2012).

Inorganic particle contents and ash values are clearly not directly linked. Particle contents are, on average, lower than ash values by a factor of six, reflecting density differences between the mineral constituents and the peat organic matter and the fact that cations organically complexed in plant tissues contribute only to the ash (Andrejko et al. 1983). In the fens, especially, groundwater likely provided additional nutrients to the mires, increasing ash contents by metal ion sorption processes.

The smallest difference between ash- and inorganic-particle content characterizes the Puścizna Mała bog (ash content higher by a factor of ca. 3) and the largest does likewise for the lowermost layers (>60 cm) of the Bagno Bruch and Bagno Mikołeska mires (ash higher by a factor of 14–37). Significantly, mineral contents are similar in the lowermost layers of all of the peat sections whereas ash values are higher in the Upper Silesian mires.

### Potential and limitations of the SEM point-counting method

The analysis provides data on the morphology and elemental composition of each counted particle. If particles such as dust particles are small, homogenization will not affect particle size in any appreciable way and, thus, size can also be measured quantitatively. Conversely, the distinction of particles lacking characteristic morphological features, but having similar chemical compositions, is impossible. For that reason, we failed to distinguish between quartz and biogenic silica—an easy task with an optical microscope. Nevertheless, inspection of a whole sample usually allows for a qualitative judgment on the role of a given particle type.

Based on chemical composition and morphology, five groups of inorganic particles may be distinguished in Puścizna Mała bog, and these with an additional two in the heavily polluted Upper Silesian mires. However, relations between the groups and their origin are complex (Table  1). Mineral matter in peat, in terms of its origin, can be classified into four fractions (Andrejko et al. 1983; Smieja-Król et al. 2010), namely, biogenic components, detrital mineral components (from rock weathering, soil dust, and volcanic ash), authigenic minerals formed in situ and anthropogenic (technogenic) components from industrial processes. In our study, feldspar was the only major mineral for which a purely detrital origin could be assigned; any contribution from industrial sources was likely to be limited. In fly ash, feldspar contents are lower than those of quartz which constitutes a major fraction (Querol et al. 1996; Vassilev and Vassileva 1996). Fly ash feldspar analyzed by Vassilev and Vassileva (1996) fell in the 20- to 30-μm size range, limiting long-range transport. No increase in feldspar contents occurs over the depth range dominated by SAP in the Upper Silesian mires.

**Table 1:** Origin of the groups of inorganic particles distinguished based on their chemical composition and morphology (detrital (D), anthropogenic (An), biogenic (B), and authigenic (Au))

Groups of inorganic particles	Minerals/phases	Origin
NS aluminosilicate	Mainly layered aluminosilicates (clays, mica)	D > An
Feldspar	K feldspar = plagioclase (mainly albite) in PM; K feldspar > plagioclase (mainly albite) in BM and BB	D
SAP	Glassy > mullite > spinel	An
Silica	Quartz > opaline silica	D > B and An
Fe(hydro)oxides	That is, magnetite, hematite, and goethite	D and An
Barite	Barite	An and Au
ZnS	Sphalerite, wurzite, nanocrystaline, or amorphic	Au > An

If obvious, the relations in amount are also indicated

The spheroidal inorganic particles (SAP and Fe spherules) probably originate entirely from industrial activities, mainly coal combustion. Cosmic spherules, almost undistinguishable from anthropogenic spheroidal dust particles, are extremely rare in peat (Franzén 2006). Larger concentrations of inorganic spherules never coincide with volcanic horizons, or with charcoal concentrations (Franzén 2006); volcanic eruptions and wildfires are unlikely sources. As the spheroids occur only in upper peat layers, an anthropogenic origin is indicated.

A key advantage of the method is very low amount of sample required. This is of primary importance when attempting high-resolution reconstructions of the atmospheric deposition of mineral dust and trace elements using peat profiles (Givelet et al. 2004). The amount of sample used is limited more by the need to analyze an amount that is representative rather than by what is physically necessary to do the analysis. Conversely, although sample preparation is relatively simple, the analysis is time consuming for low-ash samples. Thus, the method is especially recommended for peat layers in which the anthropogenic character of the particles is observable, or for organic materials with ash contents > ~4 %. The SEM point-counting method conducted on bulk samples is also useful for minerals prone, during extraction, to oxidation, dehydration, re-crystallization or dissolution, e.g., sulfides, clays, gypsum, opaline silica, or glassy SAP.

### Particle size—local or distant sources

Sizes of dust-deposited particles depend, *inter alia*, on distance from source, local climate conditions and surface relief (Lawrence and Neff 2009). Furthermore, burial in peat leads to modification in particle size, disintegration, and dissolution over time (Le Roux and Shotyk 2006). Nevertheless, larger grains point to a local origin. Such is indicated when a substantial portion of grains with between 10 and 60 % of the total mass are particles of >20 μm in diameter (Lawrence and Neff 2009).

At Bagno Mikołeska, the maximum mean size (20 μm) of the grains seen at 47 cm depth in the BM1 core coincides with increased quartz and the highest ash content. Here, the sudden increase in the abundance of larger grains is attributable to mobilization of dunes surrounding the core site due to forest managing.

The observed differences in SAP size between the peats relate to distance from fly-ash emission sources. Puścizna Mała bog, hosting the smallest SAP, is located far from industry centers. The average SAP size in the Upper Silesian mires is distinctly greater, with the largest grains present in Bagno Bruch, located nearest to emission sources. Spheroidal Fe oxides in the Puścizna Mała peats are even smaller, reflecting their higher density and shorter residence time in the atmosphere. Before any distance-related size segregation occurs, the sizes of magnetic spherules range from a few to hundreds of micrometers (Magiera et al. 2011). Interestingly, the introduction of particle control systems in power stations is not reflected in profile grain-size distributions. The generally uniform SAP size with depth seems to confirm that distance is the main size-controlling factor.

Large aggregates of porous aluminosilicates (Fig.3e) (Fiałkiewicz-Kozieł et al. 2011) occur in low numbers in all of the peats examined. Nearby coal-based domestic central-heating units are the likely sources.

### Inorganic ash spheres as tracers of atmospheric pollution

Spheroidal anthropogenic particles are unambiguous indicators of industrial activity. These particles are found worldwide in lake sediments (Rose 1996). To date, mostly spheroidal carbonaceous fly-ash particles (CPS) have been used as spatial- and historical pollution tracers due to their ease of extraction by acid digestion (Rose 1996).

In the Upper Silesia industrial region, locally sourced bituminous coal is the main fuel for power generation. Regardless of boiler type, carbonaceous matter constitutes a minor fraction (4–14 %) in fly ash. The proportion of CPS is even smaller—accounting for 3–20 % of carbonaceous matter, most of which is of irregular shape (Misz 2002, 2004). Regardless of feed coal used, inorganic ash spheres (IAS) dominate in most fly ashes.

The IAS in the peats are of two kinds—SAP and Fe-rich spherical particles (magnetic spherules). Here, we confirm the observation of Williams (1992) that Fe oxides are unstable in the peat environment and that their occurrence in peat depends on the water table. In the Bagno Mikołeska poor fen where the water table is constantly high and seasonally above the peat surface, Fe oxides are below the detection limit. Fe oxides occur only in the uppermost layer at Bagno Bruch where the poor fen endures dynamic water-table fluctuations and the maximum level at the surface is not typical. In the Puścizna Mała peat bog, on the other hand, Fe-rich particles track concentrations of SAP, and contents there are much higher than in the Upper Silesian mires even though concentrations of SAP in the mires are significantly higher. Thus, the Fe-rich spheroidal particles, unmistakable in their origin, are the better indicators of redox conditions and a long-lasting average water table. Low water table episodes are further confirmed by authigenic gypsum in the Bagno Bruch and Puścizna Mała cores.

### Ti content in SAP

Ti enrichment up to few weight percent is typical of the SAP composition whereas, in nondescript aluminosilcates, Ti contents fall below the EDS detection limit. Rare spherical Ti oxides (Fig. 3g, h) confirm some Ti oxides to be anthropogenic.

SAP particles collected from the Upper Silesia atmosphere contain between 1.45 and 7.81 % TiO_2_ (Jabłońska 2003). TiO_2_ contents are typically ~1 % in coal fly ash (e.g., Martinez-Tarazona and Spears 1996; Vassilev et al. 2001; Chen et al. 2004)—a value higher than the average concentration estimated for the upper continental crust of 0.54–0.64 % (Rudnick and Gao 2003). Furthermore, Goodarzi (2006) has noted that Ti contents increase with decreasing fly-ash grain size and Chen et al. (2004) have identified several Ti phases in ultra-fine fly ash particles.

The anthropogenic contribution of Ti in peat profiles merits further evaluation. Deemed a conservative lithogenic element, Ti is often used as a proxy for soil-dust contributions to trace-element concentrations in peat (e.g., Martinez Cortizas et al. 2002; Weiss et al. 2002; Farmer et al. 2009; Pontevedra-Pombal et al. 2013). Although Ti contents closely follow ash contents (e.g., Fiałkiewicz-Kozieł et al. 2011; Pontevedra-Pombal et al. 2013), where fly ash is an important component, Ti cannot proxy for soil dust in peat.

## Conclusions

The SEM point counting method described enables the quantitative determination of anthropogenic particles and minerals in peats with ash contents > ~4 %. During analysis, data on morphology, elemental composition, and particle size is obtained. In a mineralogical context, the type of peatland is of minor significance as long as the mires experience no surface water input. The critical factors influencing the mineralogy are distance from pollution source and water conditions within the peatland. SAP, as they are of common occurrence and easily characterized, are the best markers of industrial pollution. The presence of SAP, in modifying the Ti distribution in peats, questions the use of this element as a proxy for lithogenic constituents.
